# Carbon dioxide laser treatment for Darier disease

**DOI:** 10.1016/j.jdin.2024.10.004

**Published:** 2024-11-13

**Authors:** Hasina Maredia, Christian Baum, Randall Roenigk, Addison Demer

**Affiliations:** Department of Dermatology, Mayo Clinic, Rochester, Minnesota

**Keywords:** CO2 laser, Darier disease, general dermatology, management, surgical dermatology, therapy

*To the Editor:* Darier disease is a chronic disease that can significantly impair quality of life due to discomfort, malodor, and disfigurement from the thick, hyperkeratotic plaques.^1^, ^2^, ^3^, ^4^ Treatment options are limited with varying success. Oral retinoids have been considered the standard treatment for Darier disease but may be contraindicated or ineffective.^1^, ^2^, ^3^, ^4^ In this study, we evaluated the utility of carbon dioxide (CO_2_) laser as a treatment modality for Darier disease.

A retrospective case series was performed of 12 patients with Darier disease who were treated with CO_2_ laser at Mayo Clinic, Rochester, MN. Weck 525 CO_2_ Surgical Laser (Weck Instruments, Inc) or Lumenis UltraPulse CO_2_ (Lumenis, Inc) was used. Settings varied by treatment; in most cases a 125-mm handpiece was used in defocused fashion in continuous wave mode, with power ranging from 10 to 60 watts and between 1 and 4 passes (Table I). Gentle curettage was used between passes with an endpoint of yellow-white, firm, dermal tissue.

**Table I:** Clinical characteristics, CO_2_ laser treatment settings, and outcomes

Case	Sex	Age∗ (y)	Tx #	Site	Anaesthesia	Settings	BSA (cm^2^)	Outcomes	Prior/other tx†
1	M	18	1	forehead	Local, CS	UltraPulse fractionated pattern-generator, 300 mJ, 60 W, 9 mm spot	90	Residual disease after first treatment. Complete response after second treatment, with no recurrence at 14Y F/U	Topicals
		18	2	forehead	Local, CS	Weck 20 W (1-3 passes) suprapulse with 7 W to blend	40		
2	M	25	1	buttocks	Local, CS	Weck 25 W (6 passes) to one side, 60 W (4 passes) to the other side	150	No evidence of recurrence at 15Y F/U	Oral retinoid, topicals
		30	2	buttocks	Local, CS	Weck 60 W (2-4 passes)	145	No recurrence at 10Y F/U	
3	M	36	1	forearm	Local, CS	Weck 10 W (1st pass), 20 W (2nd pass)	315	No F/U	Oral retinoid
4	M	37	1	buttocks, hip	Local	Weck 20 W (1st pass), 10 W (2nd pass)	426	No recurrence at 12Y F/U, 4-wk recovery, with 1 wk of pain	Oral retinoid, topicals
		49	2	inguinal folds, buttock, legs	General	Weck 45 W (1-2 passes)	550	No recurrence at 3M F/U, activity in periphery, >6-wk healing time	
5	F	49	1	breast, abdomen	Local	Weck 10 W (1 pass)	18	No recurrence at 12Y F/U, pain/scar limited further tx	Topicals
6	F	47	1	groin	Local	Weck 10 W (1st pass), 7 W (2nd-3rd passes)	40	No recurrence at 9Y F/U, pain/scar limited further tx	Oral retinoid
7	F	51	1	ears, neck, gluteal fold	Local, CS	Weck 25-35 W (2 passes)	274	No recurrence at 18Y F/U	Oral retinoid, topicals
		57	2	face, neck, ears	General	UltraPulse fractionated, pattern generator, 225 J/cm^2^, density 5; face/neck: 20 W (1-2 passes); eyelids/ears: 5 W (1 pass)	200	No recurrence at 12Y F/U	
	F	63	3	ear, neck, inframammary fold, lower back, suprapubic	General	Weck 50 W (2 passes), except ears: 25 W (2 passes)	1528	No recurrence at 6Y F/U	
		69	4	lower back	General	Weck 10 W (2 passes)	560	No F/U (patient death due to co-morbidities)	
8	F	71	1	forehead, upper back	Local	Weck 15-20 W (2 passes)	52	>6-wk healing time, possible koebnerization	Topicals
9	F	23	1	leg	Local	Weck 20 W, superpulse 2 mode (1st pass), 10 W superpulse 1 mode (2nd-3rd passes)	42	Residual disease. F/U 13Y later, with severe disease and skin grafting limiting objective assessment.	Oral retinoid
10	M	43	1	ear, buttock	Local	Weck, ear: 20 W, buttock: 40 W (3 passes)	19	No recurrence at 4M F/U	Oral retinoid
		43	2	lower back, buttocks	Local	Weck 60 W (1st pass), 25-60 W (2nd pass)	728	No F/U (patient death due to co-morbidities)	
11	M	76	1	back	Local	Weck 60 W (2 passes), 45 W (3rd pass)	520	No recurrence at 1Y F/U, new minor activity in periphery	Oral retinoid, topicals
		77	2	chest, arm	General	Weck 60 W (2-3 passes)	537	No F/U (recommended as needed)	
12	F	57	1	thigh/buttock, popliteal fossa	Local, CS	Weck 50 W (1st pass), 15-20 W (2nd pass)	306	No recurrence at 15Y F/U	Oral retinoid, prior effective CO2 treatment
		72	2	lower back, gluteal cleft, buttock	General	Weck 45 W (1-2 passes)	182	No F/U	

*BSA*, Body surface area; *CS*, conscious sedation; *F/U*, follow up; *M*, month; *Tx*, treatment; *Y*, year.

∗ Age at time of treatment.

† Topical treatments included retinoid, antibacterial, steroids, or calcineurin inhibitor.

Among the 12 cases, a total of 21 treatments were performed; 19 were ablative and 2 were fractionated. Average age at treatment was 47 years (range 18-76, SD 17). Nine patients had disease refractory to oral retinoids. Anesthesia included local only (*n* = 8), local with conscious sedation (*n* = 7), and general anesthesia (*n* = 6). Average body surface area treated per procedure was 320 cm^2^ (range 18-1528, SD = 342). Sites included extremities (*n* = 9), buttocks (*n* = 8), trunk (*n* = 8), face (*n* = 8), and intertriginous areas (*n* = 5).

Persistent or recurrent disease occurred after treatment for 2 cases. For one case, fractionated laser was used over the forehead with recurrence, but after changing to ablative laser with deeper dermal penetration, there was no recurrence. For the second, ablative settings were used on the legs, but the patient was lost to follow up for 13 years and had significant disease requiring grafting, limiting assessment. For the other 18 treatments among the remaining 10 cases, no recurrence occurred in treated areas. There was 1 case in which there was new, mild activity to the periphery of treated areas, a known phenomenon following ablative laser (Fig 1). Adverse effects included scar formation in all cases, pain, possible koebnerization, and wound healing time greater than 6 weeks.

**Fig 1:**
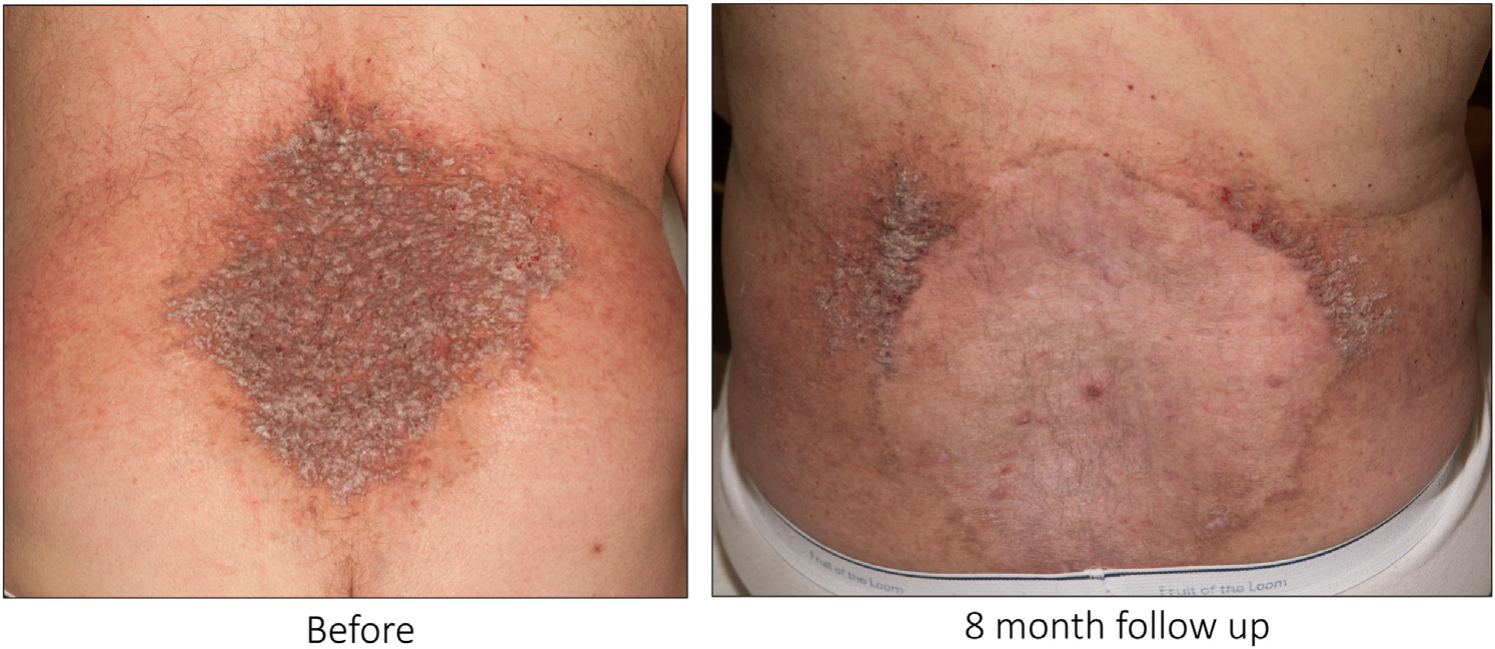
Darier disease on the lower back treated with CO_2_ laser. (*left*) A patient with Darier disease was refractory to treatment with daily isotretinoin. Ablative CO_2_ laser was used to treat Darier disease over the lower back using a 125 mm lens with 60 watts in 2 passes and 45 watts in one pass. (*right*) After 8 months, the treated areas were well-healed without residual activity or recurrence. There was new activity in the periphery of the treated area, which is a known phenomenon after ablative treatment. The patient was satisfied with his results and preferred to defer additional treatment to the periphery.

In summary, CO_2_ laser provided long-term improvement in a majority of cases, out as far as 15-year follow up. Long-term results appear to be associated with formation of ideally mild-to-moderate scarring.^1^, ^2^, ^3^, ^4^ Patients should be counseled extensively on the expected scar, pain, and possibility of prolonged healing beyond 6 weeks. Due to these factors, CO_2_ laser is best selected as an adjunctive treatment option for persistent areas after optimization of medical treatment. Limitations to the study are the number of cases, but this study doubles the number of reported cases of Darier disease treated with CO_2_ laser in the literature. Additional studies to understand the biological changes induced by successful CO_2_ laser treatment on Darier disease are warranted to help refine future novel therapies.

## Conflicts of interest

None disclosed.
